# Reactive Eccrine Syringofibroadenoma on the Heel, Clinically Mimicking Squamous Cell Carcinoma

**DOI:** 10.1155/2019/4735739

**Published:** 2019-12-04

**Authors:** Yuri Sugita, Teruhiko Makino, Kotaro Matsui, Tadamichi Shimizu

**Affiliations:** Department of Dermatology, Graduate School of Medicine and Pharmaceutical Sciences, University of Toyama, Toyama, 930-0194, Japan

## Abstract

The authors present a case of eccrine syringofibroadenoma that clinically mimicked squamous cell carcinoma and briefly comment on the current knowledge about its clinical and histopathological features and therapeutic options.

## 1. Introduction

Eccrine syringofibroadenoma (ESFA) is a rare benign skin disease that originates from the eccrine ducts. First described by Mascaro in 1963 [1], ESFA is histologically characterized by anastomosing strands of epithelial cells embedded in a fibro-vascular stroma. However, despite its distinct histological manifestation, the clinical appearance of ESFA is nonspecific and variable. We herein report a case of reactive ESFA with hyperkeratosis and hematomas on the heel, clinically mimicking squamous cell carcinoma.

## 2. Case Presentation

**Figure 1:**
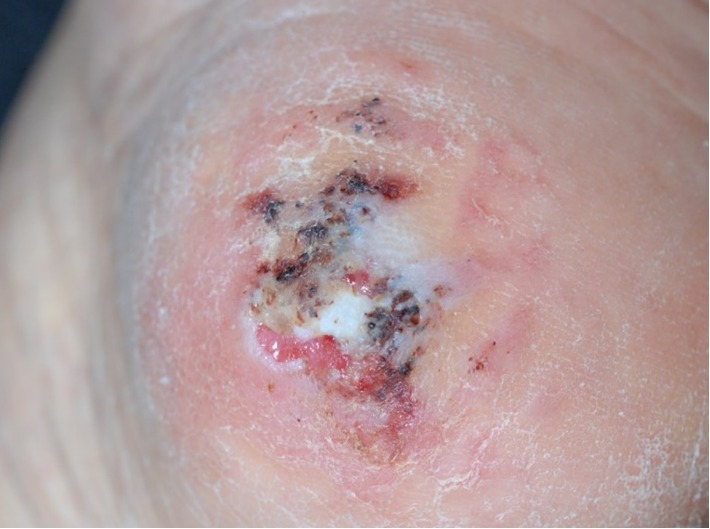
Clinical findings of the patient. Erythema plaque with multiple brown macules, ulcer, and hyperkeratinization on the left heel.

A 90-year-old Japanese woman presented with a 4-year history of hyperkeratotic lesions on her left heel that had not been improved by topical treatment with salicylic acid petrolatum ointment or 10% urea cream. There was no history of trauma, but she gradually found it hard to walk because of her left heel pain. The patient had been taking clopidogrel, aspirin, azosemide, metformin hydrochloride, and alogliptin benzoate for the treatment of chronic heart failure, angina, and diabetes mellitus, and all of these symptoms had been well controlled. At the first visit, a clinical examination revealed an erythematous plaque with multiple brown macules, ulcer, and hyperkeratosis (Figure 1).

**Figure 2:**
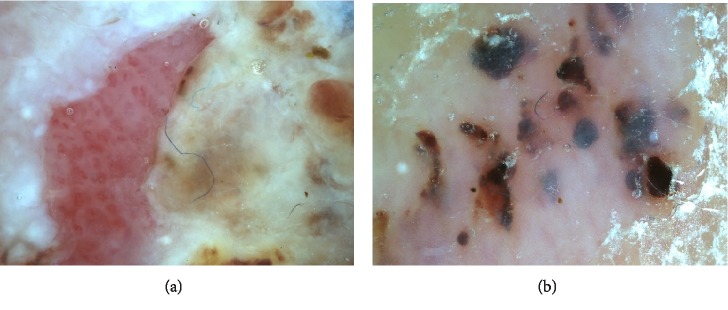
(a) Dermoscopy of the ulcer lesion. Glomerular vessels with regular arrangement in the ulcer area. (b) Dermoscopy of the erythematous plaque. There were features of intracorneal hemorrhaging.

Dermoscopy demonstrated glomerular vessels with regular arrangement in the ulcer area (Figure 2(a)) and red-black homogeneous areas in the erythematous area (Figure 2(b)). She had no particular family history. Although we recommended that she undergo a skin biopsy to diagnose the skin lesion, the patient and her family expressed a strong desire to treat the lesion through a single surgical operation because she was at an advanced age, and the possibility of squamous cell carcinoma could not be completely excluded. Therefore, the skin lesion was totally excised.

**Figure 3:**
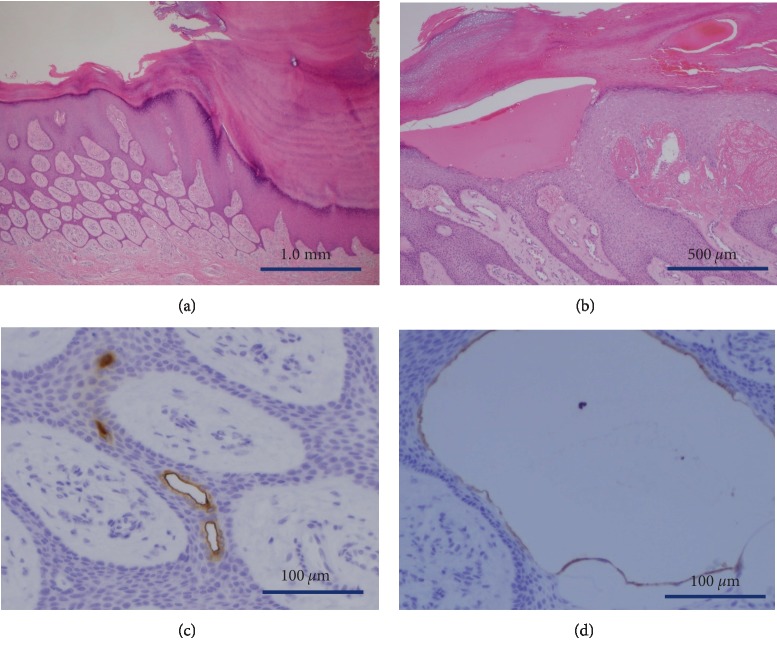
(a) Hyperkeratosis with thin anastomosing strands of cuboidal cells extending from the epidermis to the upper dermis (H&E stain, original magnification × 2). (b) Hematomas in the intracorneal region and upper dermis (H&E stain, original magnification × 4). (c) The cells of the ductal and cystic structures (d) were positive for CEA (original magnification × 20).

The histopathological examination revealed hyperkeratosis and thin anastomosing strands of cuboidal cells extending from the epidermis to the upper dermis (Figure 3(a)). Small ductal structures and cystic changes were observed within the interconnected strands of cells. Hematomas were found in the intracorneal region and upper dermis (Figure 3(b)). No mitotic or dysplastic cells were observed. The cells of the ductal and cystic structures were positive for carcinoembryonic antigen (CEA) (Figures 3(c) and 3(d)). Based on these findings, the lesion was diagnosed as a reactive ESFA.

## 3. Discussion

ESFA is commonly classified into five subtypes: (i) solitary ESFA, (ii) multiple ESFA associated with hidrotic ectodermal dysplasia (Schöpf syndrome), (iii) multiple ESFA without associated cutaneous findings (eccrine syringofibroadenomatosis), (iv) non-familial unilateral linear ESFA (nevoid ES), and (v) reactive ESFA associated with an inflammatory or neoplastic process [2–4]. The present patient was diagnosed with reactive ESFA based on the characteristic histological and immunohistochemical findings.

Reactive ESFA is associated with tissue remodeling and has been found together with erosive palmoplantar lichen planus [3], bullous pemphigoid [5], burn scar [6], lepromatous leprosy [7], diabetes mellitus with polyneuropathy and chronic ulcers [8], nevus sebaceous, or squamous cell carcinoma [9]. In the present patient, both chronic stimulation and repetitive ulceration may have been associated with the development of reactive ESFA. The lesion in the present patient demonstrated unique findings, such as hyperkeratosis, ulceration, and hematomas. Although ESFA is well known to show a varied clinical appearance, no findings similar to ours have yet been reported. In addition, the clinical appearance in this case suggested the possibility of squamous cell carcinoma. Repetitive bleeding, which was enhanced by aspirin, may modify the clinical appearance.

In some subtypes of ESFA, malignant transformation into syringofibrocarcinoma or eccrine porocarcinoma has occasionally been observed [10]. However, reactive ESFA usually shows no malignant transformation; surgical excision may therefore be unnecessary for treating reactive ESFA, although the skin lesion in the present patient was totally excised. Reactive ESFA showing atypical findings may clinically resemble squamous cell carcinoma; therefore, physicians should consider reactive ESFA as a disease that should be distinguished from skin malignancy.

## Data Availability

The data used to support the findings of this study are available from the corresponding author upon request.
